# Italian adaptation and validation of the International Cognitive Ability Resource 16 sample test

**DOI:** 10.3389/fpsyg.2026.1998035

**Published:** 2026-10-14

**Authors:** Eva Tolomeo, Leognano Ceraudo, Marco Balducci, Marco Tullio Liuzza

**Affiliations:** 1 Department of Health Sciences, University “Magna Graecia”, Catanzaro, Italy; 2 INVEST Research Flagship Center, University of Turku, Turku, Finland; 3 Department of Developmental Psychology and Socialisation, University of Padova, Padova, Italy

**Keywords:** cognitive abilities, cognitive reflection test, G factor, ICAR-16, intelligence, Italy, validation

## Abstract

The International Cognitive Ability Resource 16 (ICAR-16) is a public-domain measure of cognitive ability. To date, no Italian validation exists. We translated and validated the ICAR-16 in a sample of 486 Italian adults (66.4% female), who completed the ICAR-16 and the long version of the Cognitive Reflection Test (CRT-Long) online. Confirmatory factor analyses supported a second-order latent variable (SOLV) structure (CFI = 0.98; TLI = 0.98). The ICAR-16 showed strong associations with the CRT-Long at both the observed and latent levels, providing partial evidence of convergent validity. Internal consistency reliability was adequate. Measurement invariance analyses supported partial strong second-order factor invariance across sexes, indicating a largely comparable higher-order structure despite sex differences in the intercepts of three first-order cognitive domains. Differential Item Functioning (DIF) analysis provided evidence of uniform DIF for only one item (MR47), indicating a consistent difference in item responses across sexes at comparable total score levels. Overall, these findings support the Italian ICAR-16 as a reliable, freely available instrument for cognitive assessment in research.

## 1 Introduction

Human Intelligence is a psychological construct defined as the “general mental capability that involves the ability to reason, plan, solve problems, think abstractly, comprehend complex ideas, learn quickly, and learn from experience” (Gottfredson, 1997, p. 13). Human intelligence and its observed score, Intelligence Quotient or IQ, have long been studied due to their strong association with real-life outcomes, including academic and job performance (Roth et al., 2015; Schmidt and Hunter, 1998), income (Zagorsky, 2007), and clinical trajectories (Fries et al., 2025).

According to the Cattell-Horn-Carroll theory (CHC; Schneider and McGrew, 2012), one of the most psychometrically supported theories, human intelligence is organized across three hierarchical strata. Stratum I represents specific cognitive tasks and processes (e.g., Working Memory); Stratum II represents broad cognitive abilities (e.g., fluid reasoning factor); while Stratum III, at the top, represents the general factor (*g*). Within this hierarchy, *g operates* as a breadth factor that accounts for the positive correlation among Stratum II cognitive abilities (Gignac and Szodorai, 2024). Scientifically, human Intelligence, so defined and modeled, is generally measured through an intelligence test (Gottfredson, 1997).

Several intelligence tests have been developed (e.g., Wechsler Scales; Raven Matrices); nevertheless, “the science of intelligence has historically been well-served by commercial measures” (Condon and Revelle, 2014, p. 52). These copyright-protected instruments have been useful in clinical and industrial applications where they offer standardized administration and ongoing validation (Condon and Revelle, 2014). However, the proprietary nature of established measures imposes financial constraints through licensing fees, limits methodological flexibility and item content transparency, and restricts data sharing. Such challenges are particularly problematic, given the field’s growing emphasis on large-scale collaborative research and open science (Liao et al., 2008; Gambardella and Hall, 2006; Revelle et al., 2020). These limitations are highlighted by the recent open science movement that advocates for transparency, accessibility, and reproducibility as fundamental pillars of scientific advancement (Nosek et al., 2015).

Accordingly, the International Cognitive Ability Resource (ICAR) was developed as a web-based and public-domain tool to measure general cognitive ability in research settings (Condon and Revelle, 2014). Unlike proprietary tools that require royalties and limit redistribution, the ICAR is maintained in the public domain. This eliminates per-administration costs and allows for unlimited redistribution of test content, removing financial and structural barriers that traditionally hinder large-scale research. Moreover, the ICAR employs rule-based automatic item generation procedures, enabling the creation of large families of test content across a wide range of difficulty levels that render unnecessary the need to restrict access to a small pool of proprietary questions and allow for a continuously expanding item bank (Revelle et al., 2020).

The ICAR initially comprised 60 items distributed across four domains: 9 Letter and Number Series items, 11 Matrix Reasoning items, 16 Verbal Reasoning items, and 24 Three-dimensional Rotation items. Then 4 items in each domain, based on item difficulty and item factor loadings (Condon and Revelle, 2014), have been selected to create a shorter version; the so-called ICAR-16. This sample test showed favorable psychometric properties, including: a clear positive manifold (positive correlations) among the items; a good overall internal consistency; a well-fitted hierarchical structure; a reliable estimation precision within average latent trait level; measurement invariance across age ranges and sex; and evidence of concurrent validity (Condon and Revelle, 2014; Young et al., 2019). Despite its established psychometric properties, the ICAR-16 still lacks robust cross-language validation, restricting its current use primarily to English-speaking samples. To the best of our knowledge, only two studies, one in Turkish (Atmaca and Baloğlu, 2023) and another in Danish (Kirkegaard and Nordbjerg, 2015), have been conducted to adapt and validate the ICAR-16 in other languages. Building on this international validation effort, the present study aims to translate and validate the ICAR-16 in an Italian adult sample.

Specifically, we evaluated the structural and convergent validity, item-level properties, internal consistency, and sex-based measurement invariance of the ICAR-16. This involved assessing model fit, associations with the CRT-Long, IRT-based item performance, reliability, and psychometric equivalence across male and female respondents. In other words, this study not only addresses the lack of validated cognitive ability measures in the public domain for Italian-speaking populations but also contributes to the growing body of evidence supporting the instrument’s psychometric robustness and cross-cultural generalizability.

## 2 Materials and methods

### 2.1 Materials and measures

#### 2.1.1 Participants

Participant recruitment relied on a non-probability sampling method (convenience sampling). Data collection commenced in March 2024 and concluded in June 2026. Participants were excluded from the sample if they: (i) did not declare they were of legal age (18 years or older); (ii) did not provide consent for data processing; and (iii) reported a citizenship other than Italian.

#### 2.1.2 Italian version of ICAR-16

The ICAR-16 consists of 16 items organized into four distinct cognitive domains: Verbal Reasoning, Letter and Number Series, Three-Dimensional Rotation, and Matrix Reasoning (Raven’s Matrix Type). Each item features multiple-choice options (ranging from four to eight), with only one correct answer. The test provides a total score calculated as the sum of correct responses across all 16 items (range 0–16). The instrument was translated from English into Italian following the International Test Commission guidelines (International Test Commission, 2017). Specifically, a forward-backward translation procedure was employed where two independent researchers translated the items into Italian, and a third researcher back-translated them into English. No discrepancies emerged between the independent translators during the forward and back-translation phases.

#### 2.1.3 Cognitive reflection test-long version

The Cognitive Reflection Test (CRT; Frederick, 2005) is a three-item scale that measures a person’s propensity to inhibit an intuitive yet incorrect response and to engage in further reflection, which can lead to a correct solution. One of the most well-known items of the CRT is: *A bat and a ball cost $1.10 in total. The bat costs $1.00 more than the ball. How much does the ball cost?______*. Although many participants would intuitively respond with 10 cents, the correct answer is 5 cents (Frederick, 2005). In the present study, we employed the six-item version of the CRT (Primi et al., 2016) to mitigate potential lack of generalizability to lower-ability participants and better discriminate between high-ability individuals. The composite score ranged from 0 to 6 and was computed as the total number of correct responses (Primi et al., 2016). Several scholars (Otero et al., 2022; Primi et al., 2016; Toplak et al., 2011) suggested that the CRT composite score correlates positively with cognitive ability test scores, indicating that CRT, ultimately, is an intelligence measure (Otero et al., 2022).

#### 2.1.4 Testing procedures

The study was conducted following approval from the Ethics Committee of the Center for Research and Psychological Intervention (CeRIP), protocol no. 150203, dated December 27, 2023. Data collection was conducted online via Google Forms accessible on both mobile devices and personal computers.

Data administration strictly adhered to the approved ethical protocol. Collection was conducted via secure online platforms, involving almost 500 voluntarily recruited adults. The unsupervised self-administration survey began with an introductory information sheet outlining the research’s primary objective: the Italian validation of the ICAR-16. Participation was contingent upon electronically signing an informed consent form, which guaranteed conditional anonymity. Responses were processed in aggregate, though participants could voluntarily provide an email address if they wished to receive feedback on their raw scores. The optional email addresses provided for feedback were structurally separated from the analytical records. Specifically, the Excel column containing these emails was removed immediately upon downloading the dataset, ensuring that the publicly shared data was fully de-identified prior to any statistical analysis. ICAR-16 items appeared in a fixed domain sequence: Verbal Reasoning; Letter and Number Series; Matrix Reasoning; and Three-Dimensional Rotation.

During the instruction phase, participants were explicitly instructed not to use any external aids (e.g., paper, pencils, etc.) to complete the tasks. To prevent missing values, response completion was mandatory for all items. The test battery followed a fixed sequence in which the ICAR-16 was systematically presented before the CRT-Long. Participants were instructed to complete the ICAR-16 within a maximum of 20 min. However, the online Google Form did not enforce an automatic time cutoff.

## 3 Data analysis

### 3.1 Descriptive analysis

We conducted all analyses using R (Version 4.3.1; R Core Team, 2023) and analyzed descriptive statistics of the main demographic variables (see Table 1).

**Table 1:** Demographic characteristics and macro-regional distribution by sex.

Variable	Total sample	Female	Male
*N*	486^1^	322^2^	163
Age, *M* (SD)	25.3 (11.2)	24.6 (10.8)	26.6 (11.7)
Age range	18–78		
Education level, *n* (%)			
Undergraduate, *n* (%)	360 (74.1%)	258 (80.1%)	101 (62.0%)
Bachelor, *n* (%)	64 (13.2%)	31 (9.6%)	33 (20.2%)
Master’s degree, *n* (%)	45 (9.3%)	24 (7.5%)	21 (12.9%)
Ph.D./Postgraduate, *n* (%)	17 (3.5%)	9 (2.8%)	8 (4.9%)
Macro-region of residence, *n* (%)			
South	269 (55.3%)	203 (63.0%)	65 (39.9%)
Center	43 (8.8%)	24 (7.5%)	19 (11.7%)
North	174 (35.8%)	95 (29.5%)	79 (48.5%)

^1^From 494 participants of the initial sample, 8 were excluded because they declared a non-Italian Nationality; ^2^One participant reported intersex as their sex and was treated as missing (NA) in some subsequent analyses. Macro-region percentages were computed among participants included in the female/male analytical comparison, *N* = 485.

Geographically, most participants came from Calabria, Veneto, Lazio, and Friuli-Venezia Giulia, while no participants were from Puglia, Basilicata, Molise, or Valle D’Aosta. Furthermore, Italian regions were aggregated into three macro-areas (North, Center, and South) based on the Nomenclature of Territorial Units for Statistics (NUTS 1) classification adopted by Eurostat (Eurostat, 2026).

### 3.2 Structural validity

We conducted a Confirmatory Factor Analysis (CFA) to examine the structural validity of the ICAR-16 using lavaan (Rosseel, 2012) and semTools (Jorgensen et al., 2025) packages. Given the categorical nature of the item responses and the expected violation of multivariate normality, we employed the robust estimator Diagonally Weighted Least Squares (DWLS). All fit indices were derived in their scaled versions to account for non-normality in the data (Li, 2016; Muthén, 1984).

We hypothesized four alternative structural models, specified according to a progression from the simplest to the most complex representation of the ICAR-16 factor structure. First, we tested a unidimensional model (Figure 1), in which a single general factor (g) directly loaded onto all 16 ICAR items, thus assuming that item responses reflect a single underlying dimension of cognitive ability. Second, we specified a bifactor model (Figure 2), in which each item loaded simultaneously onto a general factor (g) and its corresponding domain-specific factor, with the general and specific factors specified as orthogonal. Third, we tested a correlated four-factor model (Figure 3), in which the 16 items loaded onto their respective domain-specific latent factors, with the four first-order factors allowed to correlate but without specifying a higher-order general factor. Finally, we specified a Second-Order Latent Variable (SOLV) model (Figure 4), in which four first-order factors (i.e., Verbal Reasoning, Letter and Number, Matrix Reasoning, and 3D Rotation) loaded onto a higher-order general factor (g), while each first-order factor loaded onto its corresponding subset of items.

**Figure 1:**
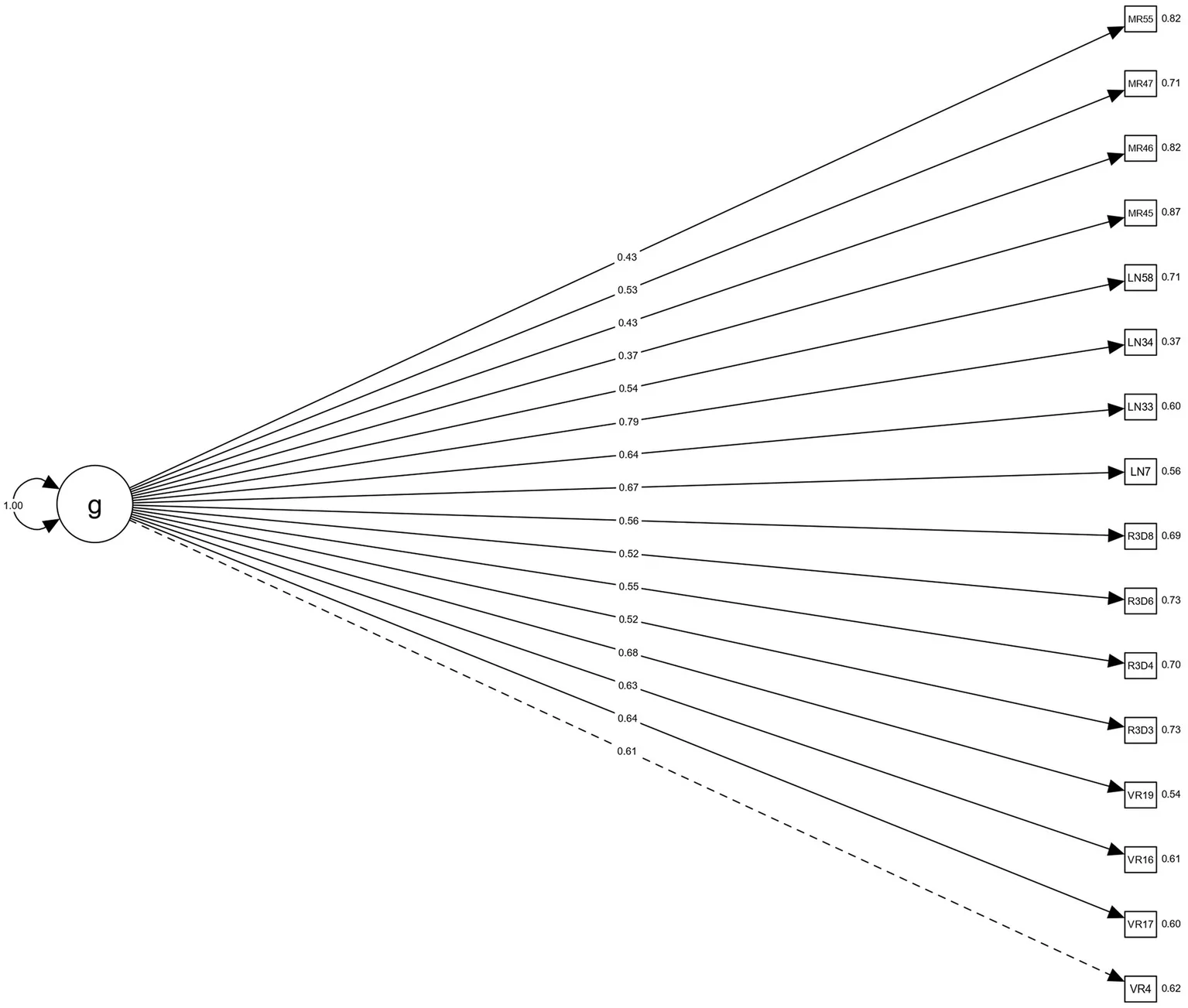
CFA: unidimensional model. The circle positioned on the left represents the general factor (*g*). The rectangles aligned on the right indicate the items. Solid single-headed arrows display the standardized factor loadings of the latent factor on each item. The dashed line pointing to the bottom item indicates the parameter fixed for model identification purposes (marker indicator). The numerical values located next to the rectangles represent the residual variances of the observed variables.

**Figure 2:**
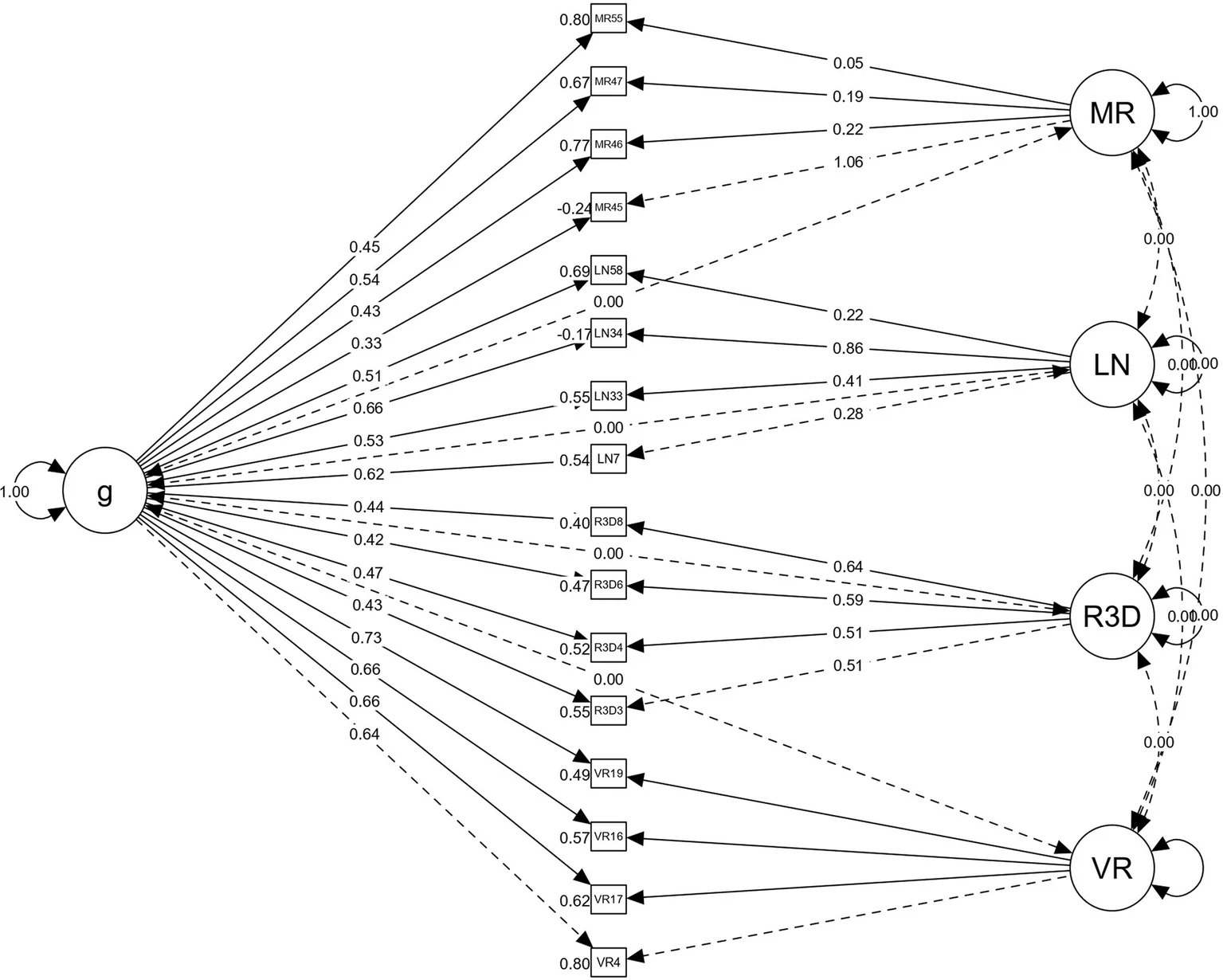
CFA: bifactor model. Circles represent the general factor (*g*) on the left, loading onto all items, and four specific group factors (matrix reasoning [MR], letter-number series [LN], 3D rotation [R3D], and verbal reasoning [VR]) positioned on the right. The specific factors are modeled as orthogonal to each other (dashed double-headed arrows indicate correlations set to 0.00). Rectangles in the central column indicate the items. Solid single-headed arrows display the standardized factor loadings, while dashed lines denote parameters fixed for model identification purposes (marker indicators). Numerical values above the rectangles represent the items’ residual variances; the diagram visually highlights the presence of Heywood cases through negative residual variance estimates (e.g., −0.24 for item MR45 and −0.17 for item LN34).

**Figure 3:**
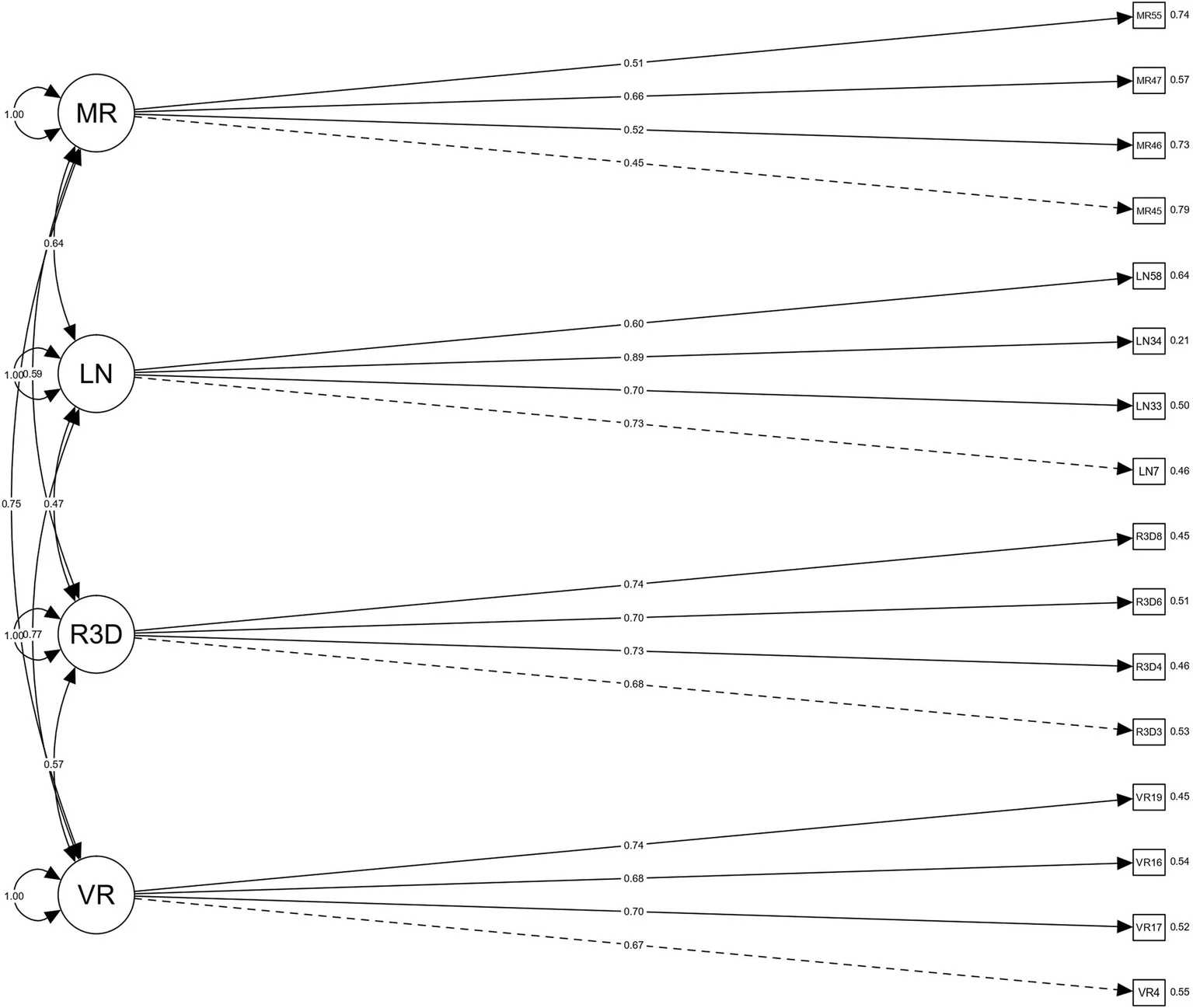
CFA: four correlated factors model. Circles represent the first-order latent variables: matrix reasoning (MR), letter-number series (LN), 3D rotation (R3D), and verbal reasoning (VR). The double-headed curved arrows on the left indicate the direct correlations among the four latent factors. Rectangles indicate the items. Single-headed arrows display the fully standardized factor loadings. Dashed lines denote the parameters fixed (typically to 1) for model identification purposes (marker indicators). Double-headed arrows and the numerical values adjacent to the rectangles represent the residual variances of the observed items.

**Figure 4:**
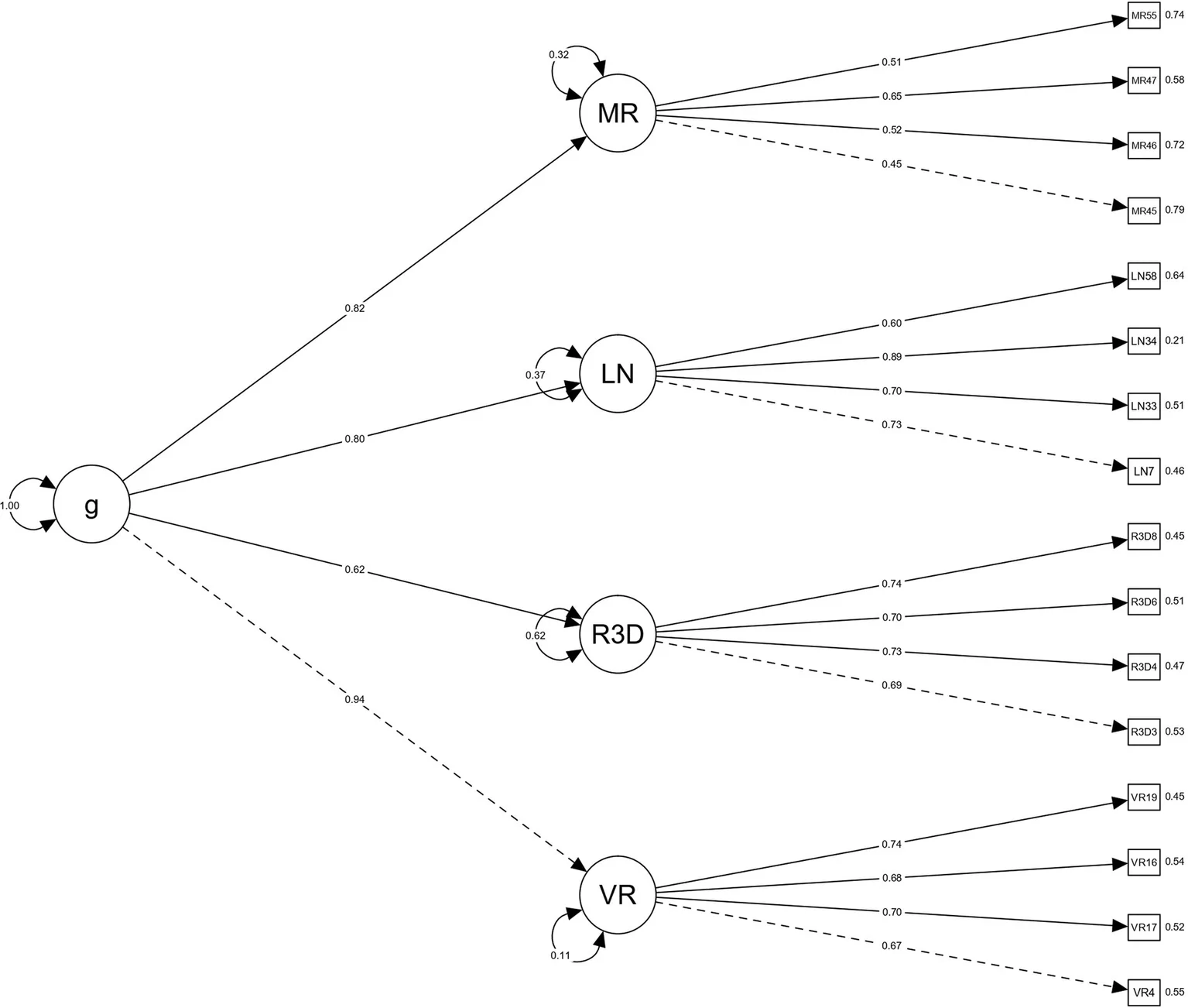
CFA: SOLV model. Circles represent the general factor (*g*) and four first-order specific factors: matrix reasoning (MR), letter-number series (LN), 3D rotation (R3D), and verbal reasoning (VR). Rectangles indicate the items. Single-headed arrows display the fully standardized factor loadings. Dashed lines represent factor loadings fixed for model identification purposes (marker indicators). Double-headed curved arrows and the values adjacent to the rectangles and first-order factors denote residual variances and disturbances.

We examined the goodness-of-fit of the models through several fit indices (Alavi et al., 2020): chi-square statistic ( χ ^2^) with its corresponding *p*-value, Tucker-Lewis Index (TLI), Comparative Fit Index (CFI), Root Mean Square Error of Approximation (RMSEA) with a 90% confidence interval (CI), and Standardized Root Mean Squared Residuals (SRMR). According to Hu and Bentler (1999), model fit was considered good if RMSEA and SRMR fell below 0.05, while values exceeding 0.90 were regarded as acceptable for the CFI and TLI. However, because robust estimators like DWLS can produce inflated fit indices and overly optimistic model interpretations (Xia and Yang, 2019), we also reported total omega3 ( ω _3_) as a measure of composite reliability, and total Average Variance Extracted (total AVE), to quantify the proportion of variance captured by the latent construct relative to measurement error (Fornell and Larcker, 1981). Finally, we compared the nested models using the Likelihood Ratio Test with Satorra and Bentler scaling correction (LRT, Satorra and Bentler, 2001), and examined comparative fit indices to determine the most parsimonious and best-fitting model.

### 3.3 Convergent validity

To test convergent validity, we first computed the Spearman correlation ( ρ ) coefficient between the ICAR-16 and CRT-Long total composite scores. Spearman’s ρ correlation was selected due to the ordinal nature of summed test scores and potential violations of the normality assumptions.

At the latent variable level, we specified five CFA models. First, we specified a unidimensional structure, where all the ICAR-16 and CRT-Long indicators loaded directly onto the *g* factor. Second, we specified a bidimensional oblique model where ICAR-16 indicators loaded directly onto a latent factor (labeled ICAR) and CRT-Long indicators loaded onto another latent factor (labeled CRT), with their factor covariance (ϕ) freely estimated. Third, we specified another bidimensional orthogonal model that constrained the factor covariance to zero (latent factors’ non-correlation). Fourth, we specified a five-dimensional model where ICAR-16 indicators loaded directly onto four latent factors (R3D, VR, LN, MR) and CRT-Long indicators loaded onto a latent factor (CRT), with their factor covariances freely estimated. Finally, we specified a second-order model where ICAR-16 indicators loaded directly onto four latent factors (R3D, VR, LN, MR), CRT-Long indicators loaded onto a latent factor (CRT), and the correlated first-order factors loaded onto a second-order general factor (*g*).

This comparative strategy evaluated the structural distinctiveness of the two scales. The three models were compared using global fit indices and the LRT test with the Satorra and Bentler scaling correction (Satorra and Bentler, 2001; Hu and Bentler, 1999). In addition, for the bidimensional oblique and the second-order model, we extracted standardized parameter estimates with 95% confidence intervals (CIs) using the “standardizedSolution” in lavaan. We then applied the 95% CI upper limit thresholds proposed by Rönkkö and Cho (2022) to evaluate convergent/discriminant validity along a continuum rather than using a rigid binary cutoff.

### 3.4 Unidimensional item response theory

The Item Response Theory (IRT) models the relationship between an individual’s latent trait level ( θ ) and their probability of responding correctly to an item (e.g., item responses; Hambleton and Swaminathan, 1985). IRT relies on three core assumptions: (1) Unidimensionality; (2) Monotonicity; (3) Local Independence (Hambleton and Swaminathan, 1985). Violations of these assumptions can bias parameters and compromise inference.

First, although dimensionality was evaluated via CFA, we also assessed the unidimensionality and the homogeneity of the scale using the psych package (Revelle, 2024). Unlike the CFA, the *u* index (1) detects subtle deviations from strict unidimensionality that may not substantially impact global CFA fit indices and (2) provides a direct and continuous quantification of the degree of unidimensionality, simplifying interpretation without requiring the integration of multiple fit metrics (Revelle and Condon, 2025). Comparison with traditional measures (i.e., hierarchical omega, comparative fit indexes, and explained common variance) showed that the unidimensionality index (*u*) has greater sensitivity to unidimensional structure and less sensitivity to the number of items in a scale (Revelle and Condon, 2025).

Second, we tested the monotonicity assumption using the mokken package (Van der Ark, 2012). Monotonicity requires that the probability of a correct item response increases monotonically with increasing levels of the latent trait.

Following assumption checks, we fitted three nested unidimensional IRT models using the mirt package (Chalmers, 2012): a one-parameter logistic (1-PL) model, a two-parameter logistic (2-PL) model, and a three-parameter logistic (3-PL) model. The 1-PL model constrains all items to have equal discrimination parameters, the 2-PL model allows item-specific discrimination parameters, and the 3-PL model additionally includes lower asymptote (guessing) parameters. We compared these models using multiple fit indexes, such as the log-likelihood values, likelihood ratio tests, the Akaike Information Criterion (AIC), the Bayesian Information Criterion (BIC), and the M2 limited-information fit statistics (Maydeu-Olivares and Joe, 2006) to evaluate absolute model fit. During the model selection phase, the Bayesian Information Criterion (BIC) was favored over other goodness-of-fit indices. Relying on the BIC is highly advantageous because it explicitly prioritizes model parsimony, penalizes unnecessary complexity, and effectively mitigates the risk of overfitting (Li et al., 2009).

Finally, we examined the assumption of local independence using Yen’s Q3 statistic (Yen, 1984), which was computed via the residuals() function in mirt with the argument type = “Q3.” Local independence assumes that item responses are conditionally independent given the latent trait. Q3 values represent correlations between item residuals after controlling for the latent trait, with absolute values exceeding 0.20 typically indicating potential local dependence (Chen and Thissen, 1997).

### 3.5 Reliability: internal consistency

We evaluated internal consistency using McDonald’s total omega ( ω *_t_*), hierarchical omega ( ω *_h_*), and Cronbach’s alpha ( α ) using the psych package (Revelle, 2025). Cronbach’s α assumes *tau* equivalence across items (Cortina, 1993), whereas ω *_t_* and ω *_h_* provide robust reliability estimates when the *tau* equivalence of items is violated (McDonald, 1999; Revelle and Zinbarg, 2009). Specifically, ω *_t_* represents the proportion of variance explained by all common factors (general and group), while (McDonald, 1999) ω *_h_* quantifies the variance accounted for exclusively by the general factor (*g*). Both coefficients range from 0 to 1, and a value close to 1 corresponds to high reliability (Revelle and Zinbarg, 2009). Finally, according to the IRT framework, we used the overall Test Information Function (TIF) and the relative marginal reliability calculated via the mirt function marginal_rxx (Chalmers, 2012; Tan, 2024). Specifically, the marginal reliability integrates the conditional measurement error variance (derived from the test information function) weighted by the assumed population density distribution of the latent trait (a standard normal distribution). This yields a single reliability coefficient that represents the expected proportion of true-score variance in the estimated θ scores for the target population (McNeish and Dumas, 2025).

### 3.6 Measurement invariance across sex

Measurement invariance across sexes was examined using Multi-Group Confirmatory Factor Analysis (MG-CFA) implemented in the lavaan package in R (Rosseel, 2012). The analysis used the SOLV model to test measurement invariance at both the first-order and second-order levels.

Models were estimated using Unweighted Least Squares with Mean- and Variance-Adjusted statistics (ULSMV) with the “theta” parameterization. The ULSMV estimator is preferred when items are categorical or ordered and when groups are severely unbalanced or sample size is small (Liu et al., 2017). Females were specified as the reference group, while several increasingly restrictive models were estimated. First, configural invariance tested whether the hierarchical structure was supported across sexes. Second, first-order strong invariance was tested by simultaneously constraining item thresholds and factor loadings to equality across groups. Because the indicators were dichotomous, these parameters were constrained jointly rather than using conventional metric and scalar invariance separately, in accordance with the Wu–Estabrook identification approach (Wu and Estabrook, 2016). Furthermore, second-order weak invariance was tested by additionally constraining second-order loadings to equality across groups. Finally, had weak second-order invariance been achieved, we would have tested for strong second-order invariance by additionally constraining the second-order intercepts to be equal across sex groups.

Model fit was evaluated using the scaled Comparative Fit Index (CFI), Root Mean Square Error of Approximation (RMSEA), and Standardized Root Mean Square Residual (SRMR). Measurement invariance was assessed primarily from changes in fit indices across successive models, with particular emphasis on the scaled CFI Specifically, a decrease smaller than approximately 0.01 was considered evidence that the additional constraints did not substantially worsen model fit (Hirschfeld and von Brachel, 2014). Scaled nested-model comparisons were also conducted using lavTestLRT().

To quantify the magnitude of potential item-level non-invariance, we additionally calculated the Difference in Mean and Covariance Structures (dMACS) effect size (Nye and Drasgow, 2011). dMACS values quantify the magnitude of measurement non-equivalence for individual items, with values of approximately 0.20, 0.40, and 0.70 interpreted as small, medium, and large effects, respectively (Nye et al., 2019). dMACS is based on the difference between the expected item scores of the two groups across the latent-trait distribution, standardized by the item’s variability (Nye and Drasgow, 2011).

As a supplementary analysis, Differential Item Functioning (DIF) was examined to determine whether individual items showed sex-related differences after controlling for overall cognitive performance.

DIF was tested using logistic regression with the difLogistic function of the difR package (Magis et al., 2010). The observed ICAR-16 total score was used as the matching variable and therefore served as an observed indicator of general cognitive ability (*g*). Females were specified as the focal group and males as the reference group. Both uniform DIF, reflecting consistent group differences across levels of overall ability, and non-uniform DIF, reflecting group differences that vary as a function of ability, were examined (Swaminathan, 1994; Zumbo, 1999). Because this procedure conditions on the observed total score rather than on a directly estimated latent *g* factor, DIF findings were interpreted as supplementary to the primary MG-CFA measurement-invariance analysis. Bonferroni-adjusted *p-*values were used to control for multiple comparisons across the 16 items.

Measurement invariance across age groups was not examined because approximately 90% of participants were between 18 and 38 years of age, resulting in insufficient age heterogeneity for meaningful multigroup comparisons.

Data, code, and Full ICAR-16 items are available at: https://doi.org/10.5281/zenodo.21787173.

## 4 Results

### 4.1 Descriptive analysis

Descriptive statistics of the Italian and non-Italian sample sizes (Condon and Revelle, 2014; Atmaca and Baloğlu, 2023; Kirkegaard and Nordbjerg, 2015) are reported in Table 2. Descriptive statistics of the CRT-Long total score of our sample are in Supplementary Table 1. In Supplementary Table 2, we reported the means and standard deviations across sex and educational levels.

**Table 2:** ICAR-16: descriptive statistics of items and total scores of Italian and non-Italian samples.

ICAR-16 total scores	Mean	Standard deviation	*N*
Condon and Revelle sample total score	7.89*	3.75*	96,958*
Danish sample total score	7.54	3.83	72
Turkish sample total score	9.09	3.46	278
Italian sample total score	7.86	3.61	486

Italian ICAR-16 items	Mean	Standard deviation	*N*
VR4	0.68	0.46	486
VR16	0.70	0.45	486
VR17	0.72	0.44	486
VR19	0.69	0.46	486
LN7	0.56	0.49	486
LN33	0.50	0.50	486
LN34	0.57	0.49	486
LN58	0.33	0.47	486
MR45	0.54	0.49	486
MR46	0.55	0.49	486
MR47	0.65	0.48	486
MR55	0.37	0.48	486
R3D3	0.19	0.39	486
R3D4	0.25	0.43	486
R3D6	0.30	0.46	486
R3D8	0.17	0.37	486

*Statistics computed based on the original study (Condon and Revelle, 2014).

### 4.2 Structural validity

A detailed visual representation of the tested models is provided in Figures 1–4. The four-correlated factors model displayed a significantly better fit compared to the unidimensional model ( △χ ^2^ (6) = 111.98, *p* < 0.001). The SOLV model yielded fit indices nearly identical to those of the four-correlated factors model, with no significant degradation ( Δχ ^2^ (2) = 2.829, *p* = 0.243, Δ CFI = − 0.001). Conversely, the bifactor model exhibited Heywood cases (e.g., negative variances for specific items and the VR specific factor; see Figure 2), rendering it unstable and uninterpretable (Kolenikov and Bollen, 2012).

Regarding internal consistency, the SOLV model achieved an excellent overall composite reliability estimate ( ω _3_ = 0.82). However, its overall Average Variance Extracted fell below the recommended benchmark (AVE_t_ = 0.46), indicating that the general intelligence factor (*g*) accounts for a relatively limited proportion of common variance across all indicators. At the subscale level, the AVE of the first-order factors reached or approximated the conventional threshold of 0.50 (Fornell and Larcker, 1981) for R3D (AVE = 0.51), LN (AVE = 0.55), and VR (AVE = 0.49). In contrast, the Matrix Reasoning factor yielded a markedly lower value (AVE = 0.29), meaning that MR items share limited common variance attributable to their domain-specific latent factor. Furthermore, the first-order factors’ reliability achieved suboptimal values (Cronbach’s α ranging from 0.47 to 0.67). This means that the individual items are relatively weak indicators when scored as separate domain subscales (see Table 3).

**Table 3:** Confirmatory factor analysis models’ fit indexes and model comparison.

Fit index/parameter	**SOLV**	**Four correlated factors**	Unidimensional
*N*. parameters	**36**	**38**	32
χ ^2^/d*f*	128.62**/100	126.034*/98	302.10***/104
Δχ ^2^	**+2.82**		+111.98***
RMSEA [90% CI]	**0.02 [0.008, 0.04]**	**0.02 [0.008, 0.04]**	0.06 [0.05, 0.07]
SRMR	**0.06**	**0.06**	0.09
CFI	**0.98**	**0.99**	0.89
TLI	**0.98**	**0.98**	0.88
ω _3_	**0.82**	**0.82**	0.80
AVE_t_	**0.45**	**0.46**	0.33

*N*: 486; *N*. parameters, number of model parameters; Models were estimated using the Diagonally Weighted Least Squares (DWLS) estimator; SOLV, Second Order Latent Variable Model; χ ^2^, Chi square statistics; RMSEA, root mean square error approximation; 90% CI, confidence interval; SRMR, standardized root mean squared residual; CFI, Comparative Fit Index; TLI, Tucker Lewis Index; ω 3, total omega3; AVE_t_, total average variance extracted; d*f*, degree of freedom; Δχ ^2^, delta Chi square; All the fit indexes are in the scaled version; ***p < 0.001; *p < 0.05; **p < 0.01; Best fitting models highlighted in bold.

### 4.3 Convergent validity

The ICAR-16 and CRT-Long total observed scores showed a strong positive correlation ( ρ = 0.62, *p* < 0.001). At the latent level, the bidimensional oblique model demonstrated adequate fit indices (see Table 4) and revealed a strong latent correlation between the overall ICAR and CRT domains (ϕicar-crt = 0.81, 95% CI: 0.75, 0.86). However, a five-dimensional oblique model (incorporating the four ICAR-16 sub-dimensions and the CRT-Long as distinct but correlated factors), demonstrated a significantly superior fit compared to the other tested models, including the bidimensional oblique model ( △χ ^2^ (9) = 133.68, *p* < 0.001). This five-factor model confirmed that the CRT-Long latent factor is highly correlated with the ICAR-16 factors, particularly with Verbal Reasoning (ϕvr-crt = 0.92, 95% CI: 0.83, 0.99). An alternative model structuring the five domains under a higher-order general intelligence factor (*g*) was uninterpretable as it produced Heywood cases (i.e., a negative residual variance for the Verbal Reasoning factor). Regarding internal consistency, the five-dimensional oblique model showed good overall composite reliability estimates ( ω _3_ = 0.89). Furthermore, its total Average Variance Extracted estimate (AVE_t_ = 0.52) suggested that slightly more than half of the variance across the factors is accounted for by common variance. The path diagrams of the bidimensional oblique and five-dimensional oblique models are provided in Supplementary Figures 1, 2 respectively.

**Table 4:** Confirmatory factor analysis models’ fit indexes and model comparison.

Fit Index / Parameter	**Five-dimensional oblique**	Bidimensional oblique	Unidimensional	Bidimensional orthogonal
*N*. parameters	**54**	45	44	44
χ ^2^/d*f*	**251.36/199**	482.97/208	587.49/209	2703.00/209
Δχ ^2^		133.68***	47.54***	3065.58***
RMSEA [90% CI]	**0.02 [0.01, 0.03]**	0.05 [0.04, 0.06]	0.06 [0.05, 0.07]	0.15 [0.15, 0.16]
SRMR	**0.05**	0.08	0.09	0.24
CFI	**0.98**	0.94	0.92	0.49
TLI	**0.98**	0.94	0.91	0.44
ω _3_	**0.89**	0.89	0.88	0.53
AVE_t_	**0.51**	0.43	0.39	0.43

*N*: 486; *N*. parameters, number of model parameters; Models were estimated using the Diagonally Weighted Least Squares (DWLS) estimator; χ ^2^, Chi square statistics; RMSEA, root mean square error approximation; 90% CI, confidence interval; SRMR, standardized root mean squared residual; CFI, Comparative Fit Index; TLI, Tucker Lewis Index; ω _3_, total omega3; AVE_t_, total average variance extracted; d*f*, degree of freedom; Δχ ^2^, delta Chi square; All the fit indexes are in the scaled version; ***: *p*-value <0.001; Best fitting model highlighted in bold.

### 4.4 Unidimensional item response theory

The scale demonstrated moderate-to-high unidimensionality and homogeneity (*u* = 0.76), confirming the presence of a dominant general latent factor (*g*) and satisfying the condition of *essential unidimensionality* (Stout, 1987). In addition, no items violate the monotonicity assumption.

Although the 3-parameter model displayed better fit indexes (M2 = 198.08; d*f* = 88) compared to the 2-parameter (M2 = 292.84; d*f* = 104) and 1-parameter model (M2 = 360.14; d*f* = 120), accurately estimating pseudo-guessing parameters in 3-parameter models requires substantially larger sample sizes (Schroeders and Gnambs, 2025). Consequently, to prioritize parsimony and parameter stability as well as interpretability, we selected the 1-parameter model for subsequent item-level evaluations (see Table 5). Under the 1-parameter model, local independence was largely supported, though three item pairs slightly exceeded the residual correlation threshold of |0.20|: LN34 − LN33 (Q3 = 0.27); R3D8 − MR55 (Q3 = − 0.21); and R3D8 − R3D6 (Q3 = 0.20).

**Table 5:** IRT models comparison.

IRT models	M2	TLI	AIC	SABIC	BIC	logLik	d*f*
**1-PL**	**360.14**	**0.89**	**8858.33**	**8874.52**	**8925.31**	**−4413.16**	
2-PL	292.84	0.90	8815.46	8847.85	8949.42	−4375.73	16
3-PL	198.08	0.93	8803.39	8851.98	9004.33	−4353.69	16

1-PL, one-parameter model; 2-PL, two-parameter model; 3-PL, three-parameter model; AIC, Akaike Information Criterion; SABIC, Sample-Size Adjusted Bayesian Information Criterion; BIC, Bayesian Information Criterion; logLik, log likelihood; df, degrees of freedom; The model with a lower BIC value is highlighted in bold.

Item difficulty parameters under the 1-parameter model (see Supplementary Table 3) range from −1.19 (VR17) to 1.91 (R3D8). More precisely, for item VR17, at a latent ability level of theta ( θ ) = −2, the probability of answering correctly is 31%; when θ is 0, the probability increases to 77%; and when θ is +2, the probability is 96%. Thus, for individuals with average latent ability ( θ = 0), the item is fairly easy. Conversely, for item R3D8, when θ is −2, the probability of answering the item correctly is 2%. When θ is 0, the probability of answering correctly is 13%, while when theta is +2, the probability of answering correctly is only 52%. For more details, the Item Probability Functions (IPFs) plot is provided in Supplementary Figure 3.

### 4.5 Reliability: internal consistency

The test achieved excellent overall estimates of internal consistency ( ω *_t_* = 0.82; α = 0.78), meaning that ICAR-16 reliably measures *g* and its cognitive facets. Nevertheless, omega hierarchical indicated that 59% of the variance in total observed scores is directly attributable to the general factor ( ω *_h_* = 0.59). The TIF analysis (Figure 5) further revealed that the ICAR-16 achieves high average precision at the average ability level ( θ = −0.1), implying that the test discriminates most reliably among individuals of average cognitive ability. The model’s marginal reliability was 0.74, which is acceptable for general research purposes (McNeish and Dumas, 2025) However, it is also likely attenuated by the incomplete unidimensionality of the scale. Such unidimensionality in turn inflates conditional standard errors of measurement across different levels of theta.

**Figure 5:**
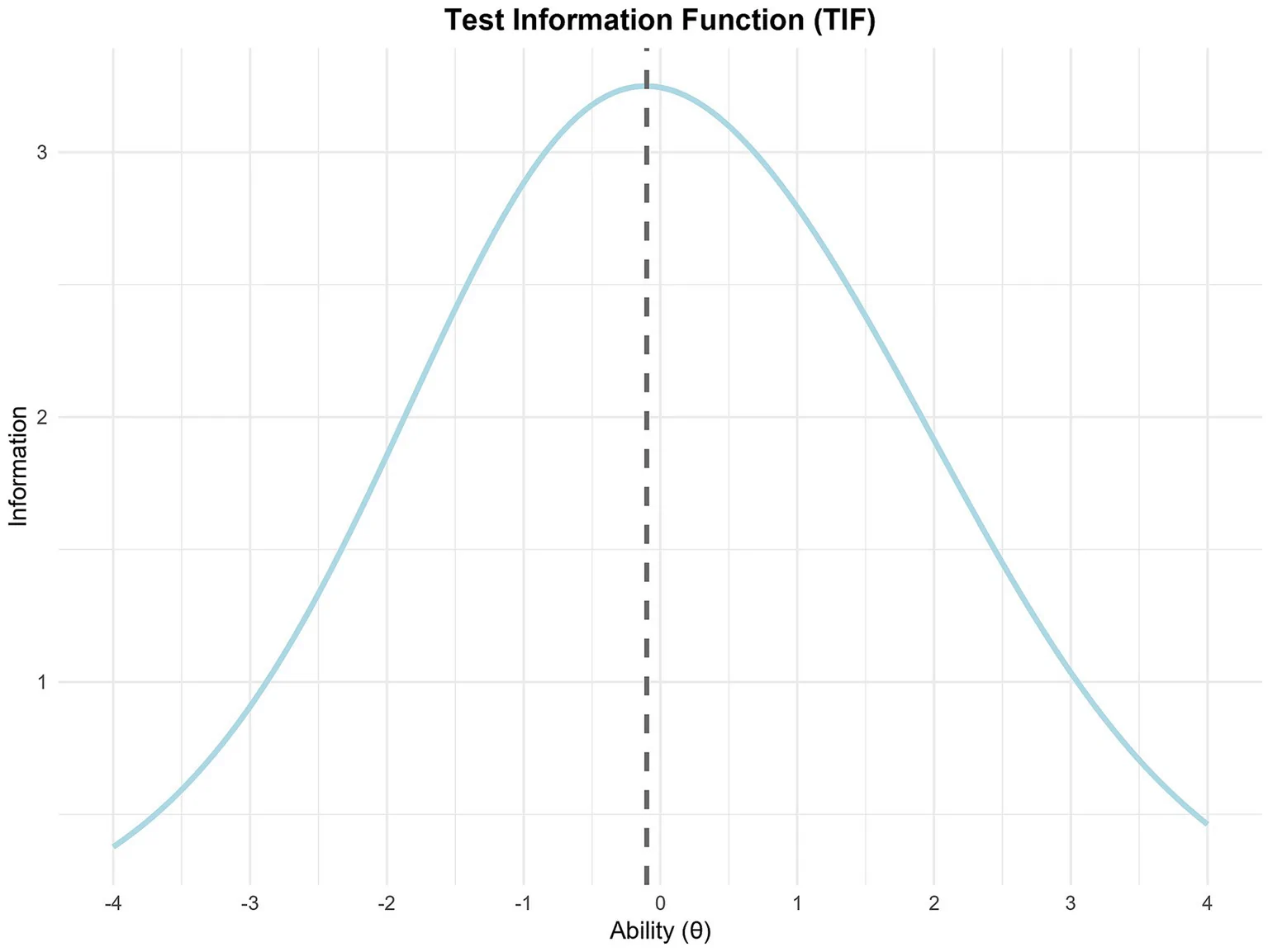
Test information function (TIF). Ability ( θ ): the underlying latent ability; Information: test accuracy across θ levels.

### 4.6 Measurement invariance across sex

Measurement invariance across sex was evaluated sequentially using a second-order CFA model (see also Table 6). First, a configural invariance model (Step 0) was estimated to assess whether the same hierarchical factor structure held across females and males. Specifically, the same four first-order factors (MR, LN, R3D, and VR), defined by the same items, and the same second-order general factor (*g*), defined by the four domains, were specified in both groups. No equality constraints were imposed on parameter estimates across sex at this stage. The configural model showed an adequate fit to the data (scaled CFI = 0.970, scaled TLI = 0.964, scaled RMSEA = 0.028, SRMR = 0.088), supporting the same overall measurement structure across groups. In Step 1 (first-order strong invariance), item factor loadings and thresholds were constrained to equality across females and males. The intercepts of the four first-order factors (MR, LN, R3D, and VR) were fixed to zero in the female reference group and freely estimated in males. These restrictions did not meaningfully worsen model fit relative to the configural model (*Δχ^2^*(10) = 13.20, *p* = 0.20; *Δ*CFI = − 0.002). In Step 2 (weak second-order invariance), equality constraints were specified for loadings, thresholds, and regressions across sex, thereby testing the invariance of the relations between the first-order domains and the second-order general factor. In the present specification, however, this step did not produce additional degrees of freedom relative to Step 1, and model fit remained unchanged (*Δχ^2^* (0) = 0; *Δ*CFI = 0). In Step 3 (full strong second-order invariance), the intercepts of all four first-order domains (MR, LN, R3D, and VR) were additionally constrained to equality across sex. The latent mean of *g* was fixed to zero in females and freely estimated in males, allowing the latent mean difference between groups to be estimated. The full strong second-order solution showed a Heywood Case (the males’ residual variance of the VR domain was negative) and was not retained. In Step 4 (partial strong second-order invariance), the intercepts of MR, LN, and R3D remained constrained to equality across sex, whereas the intercept of VR was freely estimated across groups. The latent mean of *g* remained fixed to zero in females and freely estimated in males. This model showed adequate fit (scaled CFI = 0.968, scaled TLI = 0.964, scaled RMSEA = 0.028, SRMR = 0.088) and did not worsen relative to the weak second-order invariance model (*Δχ^2^* (3) = 3.14, *p* = 0.37; *Δ*CFI = 0). Thus, partial strong second-order invariance was supported, permitting comparison of the latent mean of the general factor across sex. Item-level dMACS values for the VR domain were 0.130 for VR4, 0.213 for VR16, 0.047 for VR17, and 0.116 for VR19. Among the remaining items, only LN7 (dMACS = 0.375) and R3D4 (dMACS = 0.309) exceeded 0.30.

**Table 6:** Multi-group confirmatory factor analysis: comparison of sex measurement invariance levels.

MG-CFA levels	*N*. parameters/d*f*	*N*. equality constraints	RMSEA [90% CI]	SRMR	TLI	CFI	Δ CFI
Full configural: first + second order	72/200		0.028 [0.007, 0.041]	0.088	0.964	0.970	
**Strong first order**	**93/207**	**28**	**0.028 [0.009, 0.040]**	**0.088**	**0.963**	**0.968**	**−0.002**
**Weak second order**	**93/210**	**31**	**0.028 [0.009, 0.040]**	**0.088**	**0.963**	**0.968**	**0**
**Partial strong second order**	**90/213**		**0.028 [0.008, 0.040]**	**0.088**	**0.964**	**0.968**	**0**

*N*: 485 (322 females and 163 males); *N*. parameters/d*f*, number of model parameters and degrees of freedom of the model. *N*. equality constraints, number of model equality constraints; MG-CFA, multi-group confirmatory factor analysis; Models were estimated using Unweighted Least Squares with Mean- and Variance-Adjusted statistics (ULSMV) with the “theta” parameterization. RMSEA, root mean square error approximation; SRMR, standardized root mean squared residual; TLI, Tucker-Lewis Index; CFI, Comparative Fit Index; Δ CFI, Delta Comparative Fit Index; 90% CI, 90% confidence interval; All the fit indexes are in the scaled version; Decrements of Δ CFI below the cut-off of 0.01 are highlighted in bold; Full Configural: it tests whether the same hierarchical structure holds across males and females; Strong First Order: item factor loadings and thresholds were constrained to equality across females and males. The intercepts of the four first-order factors (MR, LN, R3D, and VR) were fixed to zero in the female reference group and freely estimated in males; Weak Second Order: equality constraints were specified for loadings, thresholds, and regressions across sex, thereby testing the invariance of the relations between the first-order domains and the second-order general factor; Partial Strong Second Order: Given that the Strong Second Order Model showed a Heywood case, we modeled a Partial Strong Second Order Model, where the intercepts of MR, LN, and R3D were constrained to equality across sex, whereas the intercept of VR was freely estimated across groups. The latent mean of *g* remained fixed to zero in females and was freely estimated in males. Fit Indexes are reported in the scaled version.

Within the IRT framework, we found only one piece of evidence of Uniform differential item functioning (MR47), after Bonferroni correction (*p* = 0.01; see Figure 6).

**Figure 6:**
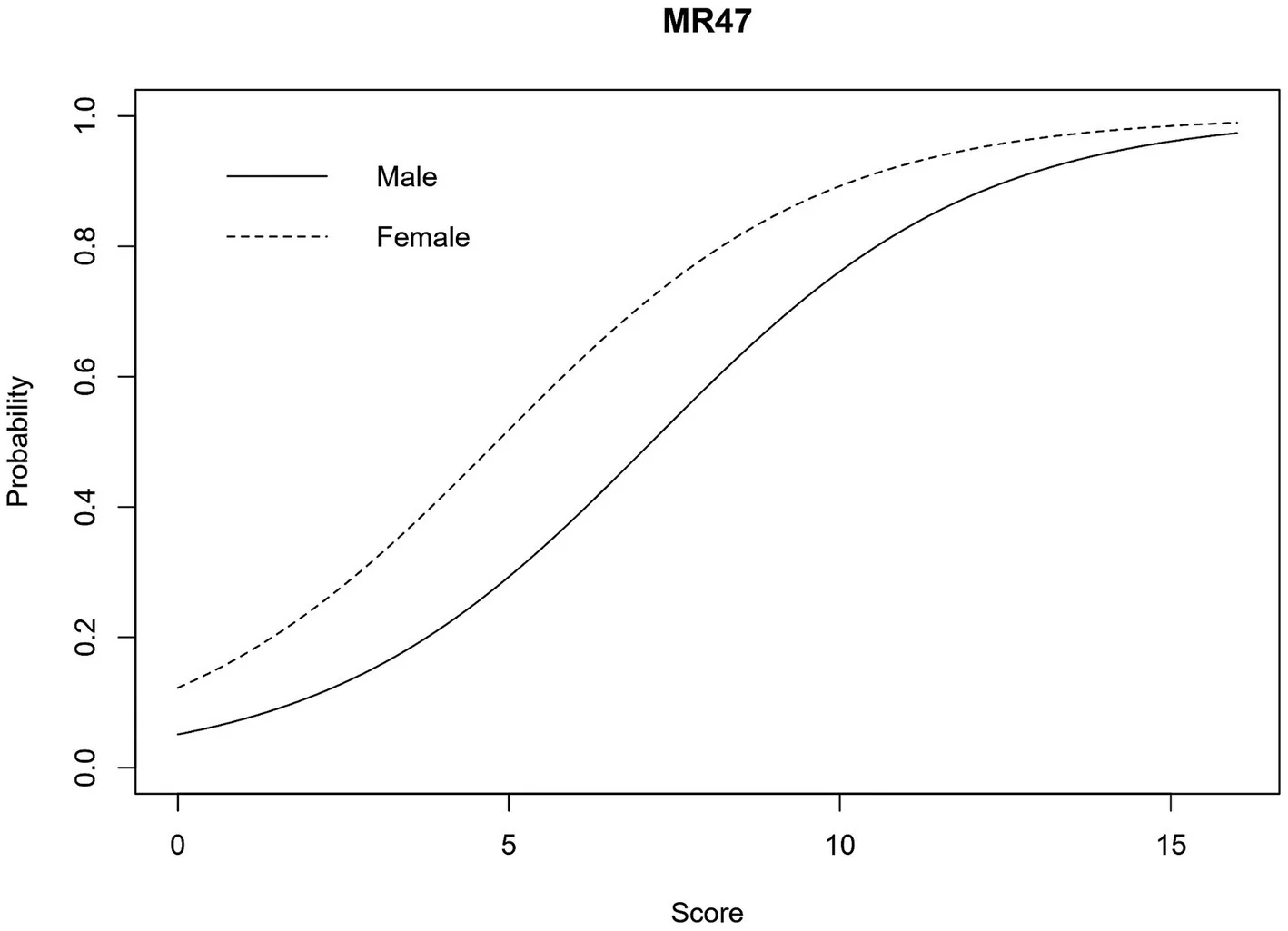
Item MR47: uniform differential item functioning. Uniform DIF: one group has a consistent advantage across all test scores.

## 5 Discussion

The present study translated and validated the ICAR-16 in an Italian-speaking sample, addressing its structural validity, convergent validity, item-level psychometric properties, internal consistency, and measurement invariance across sex.

Both the Second-Order Latent Variable (SOLV) model, which postulates a general intelligence factor (*g*) loading onto four correlated first-order factors (cognitive subdomains), and the four correlated factors model showed the best and most interpretable fit to the data. The SOLV model was preferred to the four correlated model due to theoretical reasons. This replicated the hierarchical structure proposed by Condon and Revelle (2014) and conceptually aligned with the Cattell–Horn–Carroll (CHC) theory of intelligence (Schneider and McGrew, 2012). Despite the excellent composite reliability of the SOLV model, the total Average Variance Extracted fell below the conventional threshold of 0.50 (Fornell and Larcker, 1981), indicating that the four first-order factors retain a non-trivial amount of specific variance (55%) not attributable to *g*. These results are consistent with the interpretation of the ICAR-16 as a hierarchical measure of intelligence, where specific domain skills and broad cognitive abilities (mainly fluid and visuospatial reasoning) contribute meaningfully to item responses beyond the general factor (Young and Keith, 2020). Nevertheless, ICAR-16 items do not appear to be pure or reliable indicators of their intended specific domains. Accordingly, the ICAR-16 should primarily be interpreted and used as an overall measure of intelligence, rather than using items from a single domain to assess domain-specific abilities.

The strong association between the ICAR-16 and the CRT-Long indicates substantial overlap in the cognitive abilities captured by the two instruments and is broadly consistent with previous findings linking the ICAR-16 to established measures such as the Shipley-2 and WAIS-IV (Condon and Revelle, 2014; Young and Keith, 2020). However, this overlap should be interpreted cautiously. The close association between the CRT-Long and the ICAR Verbal Reasoning domain, together with the failure of a higher-order model placing both instruments under a common *g* factor, suggests that their shared variance may reflect specific cognitive processes in addition to general cognitive ability. Accordingly, the present findings support a close relationship between the ICAR-16 and CRT-Long, but do not justify treating the latter as a pure proxy for *g* or as definitive evidence of the ICAR-16’s general-intelligence validity.

Moreover, the instrument demonstrated excellent internal consistency, in line with results from the original English-language version and subsequent translations (Condon and Revelle, 2014; Atmaca and Baloğlu, 2023). However, the hierarchical omega index was notably lower than the total omega index, indicating that the total observed score reflects not only *g* but also a non-trivial proportion of variance attributable to the four domain-specific factors. Such a pattern reinforces the interpretation of the ICAR-16 as a multifaceted cognitive battery in which subscale variance contributes meaningfully to total score variance. One possible explanation for this discrepancy is that, in samples with relatively high education levels, as in the present study, the general factor may account for less variance relative to specific cognitive abilities, as posited by *the differentiation hypothesis* (Spearman, 1927; Colom et al., 2002; Kovacs and Conway, 2016).

At the item level, the parameters of the 1-PL model range from −1.19 (VR17) to 1.91 (R3D8). This suggests that the items in the verbal reasoning domain are the least difficult, those in the three-dimensional reasoning domain are the most difficult, and items in the matrix and letter-number series reasoning domains exhibit intermediate difficulty. Overall, the Test Information Function indicates that the test is accurate across a range of cognitive ability levels, but achieves its highest accuracy at intermediate levels. This evidence is in line with previous results (Condon and Revelle, 2014) and is considerable for a cognitive ability instrument for research purposes.

Finally, the Multi-Group CFA supported strong first-order measurement invariance, indicating that item loadings and thresholds were equivalent across sexes, as well as partial strong second-order invariance. At the second-order level, factor loadings were invariant across groups, whereas the intercepts of three of the four cognitive domains (MR, LN, and R3D) were equivalent across sex, with the intercept of VR being freely estimated. Importantly, the magnitude of item-level non-invariance appeared limited: dMACS values were ≤ 0.37 across all items and fell within the negligible range according to the criteria proposed by Nye et al. (2019), suggesting that the practical impact of the observed partial non-invariance on group comparisons was marginal. Consistent with this pattern, the logistic regression-based DIF analysis identified MR47 as the only item showing significant uniform Differential Item Functioning after Bonferroni correction (adjusted *p* = 0.01).

### 5.1 Methodological limitations

The primary methodological limitations of this study concern the sample characteristics and recruitment strategy. A total of 486 participants were included in most analyses; however, for the Multigroup Confirmatory Factor Analysis (MG-CFA), one participant who reported their sex as intersex was coded as missing (NA). Although the remaining sample of 485 is generally adequate for MG-CFA (Pendergast et al., 2017), the marked numerical imbalance between female (*n* = 322) and male (*n* = 163) subgroups can lead to negative latent variable variances when testing complex models. Another limitation concerns the assessment of convergent validity. Although the CRT-Long has shown evidence of functioning as a brief measure related to the general intelligence factor, the present study did not examine the ICAR-16 against established measures of *g*. Future research should therefore assess convergent validity using comprehensive intelligence batteries that sample multiple cognitive domains and provide a more robust estimate of general cognitive ability. Furthermore, the generalizability of the instrument is restricted by the sample’s demographic homogeneity, as nearly 90% of participants were young adults predominantly localized in the Calabria region. Regarding the translation procedure, no discrepancies emerged between the independent translators during the forward and back-translation phases, allowing for a straightforward consensus on linguistic and task-rule equivalence. However, we acknowledge that neither formal cognitive interviews with independent experts nor pilot testing were conducted as part of this initial adaptation process. Moreover, Additional limitations stem from the online data collection procedures. Metadata regarding completion time and device type were not recorded, and no technical safeguards were implemented to prevent or detect duplicate or invalid submissions. Finally, the unsupervised nature of the administration poses a potential threat to internal validity (e.g., cheating or inattentive responding), which could not be controlled due to the lack of relevant tracking data.

## 6 Conclusion

The present study provides the first Italian translation and psychometric validation of the ICAR-16 sample test on a large Italian-speaking sample (*N* = 486). Overall, the results support its hierarchical structure, excellent internal consistency, and satisfactory item-level properties under the 1-PL IRT framework. Measurement invariance analyses supported partial strong second-order invariance across sexes, and the overall impact on group comparisons was found to be negligible to small in terms of practical effect size.

Taken together, these findings establish the Italian ICAR-16 as a psychometrically sound, freely available, and easily administrable tool for assessing general cognitive ability in Italian-speaking research populations. Its public-domain status makes it particularly valuable for large-scale collaborative research and for studies requiring cost-effective cognitive assessment without the licensing constraints associated with proprietary instruments. Future research should expand normative data collection across a more representative and age-diverse Italian sample, investigate test–retest reliability, and examine the convergent validity of the Italian ICAR-16 against established clinical intelligence batteries. Finally, exploring the utility of the instrument in applied settings, such as educational or occupational research, would further consolidate its role within the Italian psychometric landscape.

## Data Availability

The datasets presented in this study can be found in online repositories. The names of the repository/repositories and accession number(s) can be found at: the anonymized dataset, analysis code, and Supplementary material are publicly available on Balducci (2026) Zenodo at: https://doi.org/10.5281/zenodo.21787173. The corresponding GitHub repository is archived through Zenodo.
