# Supplementary material for: Italian adaptation and validation of the International Cognitive Ability Resource 16 sample test
Source: Front Psychol. 2026 Oct 14;17:1998035. doi: 10.3389/fpsyg.2026.1998035 (PMC13713778; doi:10.3389/fpsyg.2026.1998035)
Supplement: Supplementary file 1 [file Data_Sheet_1.docx]

***Supplementary Material***

# 1 Supplementary Figures and Tables

**Supplementary Figure 1. Convergent Validity: Bidimensional Oblique Model**

Note. Circles positioned on the left represent the two latent variables (CRT-Long and ICAR). The double-headed curved arrow connecting the two factors indicates a strong latent correlation of .81 between the two domains. Rectangles aligned on the right indicate the observed variables, grouped by their respective constructs (the 16 ICAR items loading on the first factor and the 6 CRT items loading on the second). Solid single-headed arrows display the standardized factor loadings of the latent variables on their respective items. Dashed lines denote the parameters fixed for model identification purposes (marker indicators). The numerical values located next to the rectangles represent the residual variances of the individual observed variables.

**Supplementary Figure 2. Convergent Validity: Five-dimensional Oblique Model**

Note. Circles on the left represent the first-order latent variables: Matrix Reasoning (MR), Letter-Number Series (LN), Verbal Reasoning (VR), 3D Rotation (R3D), and the Cognitive Reflection Test (CRT). Double-headed curved arrows indicate the direct correlations among all five latent factors. Rectangles aligned on the right indicate the observed variables, corresponding to items. Solid single-headed arrows display the standardized factor loadings of the latent factors on each item. Dashed lines denote the parameters fixed for model identification purposes (marker indicators). The numerical values located next to the rectangles represent the residual variances of the observed variables.

**Supplementary Figure 3. Item Probability Functions**

Note. $\theta$: Underlying ability level (the g factor in this case). P($\theta$): the probability of answering correctly to an item in function of the underlying ability level.

**Supplementary Table 1. Cognitive Reflection Test-Long total score, Items and Internal Consistency: Descriptive Statistics of Italian sample**

| **CRT-Long Total Scores and Items** | **Mean** | **Standard Deviation** | **N** |
| --- | --- | --- | --- |
| **CRT-Long** | 2.89 | 2.08 | 486 |
| **CRT1** | .37 | .48 | 486 |
| **CRT2** | .49 | .50 | 486 |
| **CRT3** | .47 | .49 | 486 |
| **CRT4** | .78 | .40 | 486 |
| **CRT5** | .34 | .47 | 486 |
| **CRT6** | .40 | .49 | 486 |
| **CRT-Long Reliability** | $\omega$**_t_** | $\alpha$ |  |
|  | .82 | .82 |  |

Note. Both $\omega$_t_ and $\alpha$ are estimated from a one-factor exploratory factor analysis using the omega function in R.

**Supplementary Table 2. ICAR-16 total score mean and standard deviation by sex and education level**

| **Education level** |  | **Total Sample** | **Female** | **Male** |
| --- | --- | --- | --- | --- |
|  |  | **Mean (SD)** | **Mean (SD)** | **Mean (SD)** |
| Undergraduate |  | 7.32 (3.54) | 6.90 (3.53) | 8.42 (3.34) |
| Bachelor |  | 9.46 (3.87) | 8.51 (3.66) | 10.36 (3.90) |
| Master’s Degree |  | 9.33 (2.90) | 8.33 (3.07) | 10.47 (2.25) |
| Ph.D./postgraduate |  | 9.11 (2.71) | 8.77 (3.46) | 9.50 (1.69) |

Note. N=485. From 494 participants of the initial sample, 8 were excluded because they declared non-Italian Nationality; One participant reported intersex as their sex and was treated as missing (NA).

**Supplementary Table 3. 1-PL model parameters**

| **ICAR-16 Items** | **Difficulty/Location (*b*)** |
| --- | --- |
| VR4 | - 0.95 |
| VR16 | - 1.05 |
| VR17 | - 1.19 |
| VR19 | - 1.03 |
| LN7 | - 0.35 |
| LN33 | - 0.03 |
| LN34 | - 0.39 |
| LN58 | 0.85 |
| MR45 | - 0.24 |
| MR46 | - 0.28 |
| MR47 | - 0.78 |
| MR55 | 0.61 |
| R3D3 | 1.74 |
| R3D4 | 1.29 |
| R3D6 | 1.01 |
| R3D8 | 1.91 |

*Note*. b: difficulty/location parameters.

Italian ICAR-16 Sample Test Items and Keys

VR.4 **Item 1.**Quale numero è un quinto di un quarto di un nono di 900?

Options: i) 2; ii) 3; iii) 4; iv) 5; v) 6; vi) 7

VR.16 **Item 2.** Luca è più alto di Matteo e Riccardo è più basso di Luca. Quale delle seguenti affermazioni è più accurata?

Options: i) Riccardo è più alto di Matteo; ii) Riccardo è più basso di Matteo; iii) Riccardo è alto quanto Matteo; iv) È impossibile dirlo

VR.17 **Item 3.** Giosuè ha 12 anni e sua sorella è tre volte più grande di lui. Quando Giosuè avrà 23 anni, quanti anni avrà sua sorella?

Options: i) 35; ii) 39; iii) 44; iv) 47; v) 53; vi) 57

VR.19 **Item 4.** Se il giorno dopo domani è due giorni prima di Giovedì, che giorno è oggi?

Options: i) Venerdì; ii) Lunedì; iii) Mercoledì; iv) Sabato; v) Martedì; vi) Domenica

LN.7 **Item 5.** Nella seguente serie alfanumerica, quale lettera viene dopo? G L N Q S

Options: i) Q;ii) R;iii) S;iv) T; v) U; vi) V

LN.33 **Item 6.** Nella seguente serie alfanumerica, quale lettera viene dopo? V Q M H F

Options: i) C;ii) D; iii) E; iv) F; v) G; vi) H

LN.34 **Item 7.** Nella seguente serie alfanumerica, quale lettera viene dopo? E F H M Q

Options: i) R; ii) S; iii) T; iv) V; v) Z; vi) A

LN.58 **Item 8.** Nella seguente serie alfanumerica, quale lettera viene dopo? Q S N P L

Options: i) J;ii) H; iii) I; iv) N; v) M; vi) L

MR.45 **Item 9.** Per favore, indichi qual è la risposta migliore per completare la figura in basso.

Options: i) A; ii) B; iii) C; iv) D; v) E; vi) F

MR.46 **Item 10.** Per favore, indichi qual è la risposta migliore per completare la figura in basso.

Options: i) A; ii) B; iii) C; iv) D; v) E; vi) F

MR.47 **Item 11.** Per favore, indichi qual è la risposta migliore per completare la figura in basso.

Options: i) A; ii) B; iii) C; iv) D; v) E; vi) F

MR.55 **Item 12.** Per favore, indichi qual è la risposta migliore per completare la figura in basso.

Options: i) A; ii) B; iii) C; iv) D; v) E; vi) F

R3D.3 **Item 13.** Tutti i cubi in basso hanno un’immagine diversa su ogni lato. Selezioni la risposta che potrebbe rappresentare una rotazione del cubo X.

Options: i) A; ii) B; iii) C; iv) D; v) E; vi) F; vii) G; viii) H

R3D.4 **Item 14.** Tutti i cubi in basso hanno un’immagine diversa su ogni lato. Selezioni la risposta che potrebbe rappresentare una rotazione del cubo X.

Options: i) A; ii) B; iii) C; iv) D; v) E; vi) F; vii) G; viii) H

R3D.6 **Item 15.** Tutti i cubi in basso hanno un’immagine diversa su ogni lato. Selezioni la risposta che potrebbe rappresentare una rotazione del cubo X.

Options: i) A; ii) B; iii) C; iv) D; v) E; vi) F; vii) G; viii) H

R3D.8 **Item 16.** Tutti i cubi in basso hanno un’immagine diversa su ogni lato. Selezioni la risposta che potrebbe rappresentare una rotazione del cubo X.

Options: i) A; ii) B; iii) C; iv) D; v) E; vi) F; vii) G; viii) H
